# Stereotypes about very old people and perceived societal appreciation in very old age

**DOI:** 10.1007/s00391-021-01971-y

**Published:** 2021-10-01

**Authors:** Marcella Reissmann, Luise Geithner, Anna Storms, Christiane Woopen

**Affiliations:** 1 Cologne Center for Ethics, Rights, Economics, and Social Sciences of Health, University of Cologne, Albertus Magnus Platz, 50923 Cologne, Germany; 2 a.r.t.e.s. Graduate School for the Humanities Cologne, University of Cologne, Cologne, Germany; 3 Research Unit Ethics, Medical Faculty, University Hospital Cologne, Cologne, Germany

**Keywords:** Stereotypes, Very old, Society, Quality of life, Mixed methods, Stereotype, Hochaltrigkeit, Gesellschaft, Lebensqualität, Mixed-Methods

## Abstract

**Background:**

People in very old age (VOA) are expected to be confronted with particularly negative stereotypes. These influence societally shared behavior towards and judgements about them. Such external evaluations of individuals’ lives are considered a crucial part of their quality of life (QoL).

**Objective:**

The present study elaborated a) the societal appreciation perceived by people in VOA and b) the stereotypes about people in VOA held by stakeholders from key societal areas. The aim was to discuss possible connections between these external standards and individual life results.

**Material and methods:**

A parallel mixed methods design was employed. Cross-sectional data from a representative survey of people aged 80 years and older (*n* = 1863) were analyzed by means of χ^2^-tests and Kruskal-Wallis tests to examine differences in perceived societal appreciation (PSA) by characteristics of the person, their biography, and current lifestyle. Linear regression models were used to investigate the impact of these characteristics on PSA. Regarding stereotypes about people in VOA, semi-structured interviews with stakeholders from key societal areas (*n* = 22) were analyzed using qualitative content analysis. The quantitative and qualitative findings were juxtaposed for comparison.

**Results:**

PSA was predicted by health-related variables and productive activities. Several societal stakeholders highlighted that age-related losses pose challenges on very old individuals, their families, and society, whereas remaining potentials in VOA can and should be used for the benefit of others; however, stakeholders‘ perceptions differed by the extent of their professional contact with (very) old people. Different pathways were proposed through which the observed stereotypes and determinants of PSA might be connected (e.g., stereotype internalization).

**Conclusion:**

Our study illustrates the relevance of external standards for individual QoL and highlights the need for a normative perspective in the discussion about QoL and its enhancement.

**Supplementary Information:**

The online version of this article (10.1007/s00391-021-01971-y) contains supplementary material, which is available to authorized users. The article and the supplementary material are available in the electronic full-text archive at https://www.springermedizin.de/zeitschrift-fuer-gerontologie-und-geriatrie. You will find the supplementary material at the end of the article.

## Abstract (de)

**Hintergrund:**

Stereotype über hochaltrige Menschen gelten als besonders negativ. Sie beeinflussen das gesellschaftlich geteilte Verhalten Hochaltrigen (HA) gegenüber und Urteile über diese. Solche externen Bewertungen stellen einen wichtigen Teil individueller Lebensqualität dar.

**Ziele der Arbeit:**

Die Studie untersuchte a) die von HA empfundene gesellschaftliche Wertschätzung (EGW) und b) die Stereotype über HA von Vertretern zentraler Gesellschaftsbereiche. Ziel war es, mögliche Verbindungen zwischen diesen externalen Standards und individuellen Lebensqualitätsresultaten (LQR) zu diskutieren.

**Material und Methoden:**

Es wurde ein paralleler Mixed-Methods-Ansatz verfolgt. Querschnittsdaten einer repräsentativen Befragung von Menschen ab 80 Jahren (*n* = 1863) wurden Chi^2^- und Kruskal-Wallis-Tests unterzogen, um Unterschiede in EGW nach Merkmalen der Person, ihrer Biographie und ihres aktuellen Lebensstils zu analysieren. Der Einfluss dieser Merkmale auf EGW wurde mit linearen Regressionsmodellen abgeschätzt. Semistrukturierte Interviews mit Stakeholdern zentraler Gesellschaftsbereiche (*n* = 22) wurden mittels qualitativer Inhaltsanalyse hinsichtlich zum Ausdruck kommender Stereotype über HA untersucht. Die quantitativen und qualitativen Ergebnisse wurden einander gegenübergestellt.

**Ergebnisse:**

EGW konnte durch gesundheitsbezogene Variablen und produktive Aktivitäten vorhergesagt werden. Einige der Stakeholder betonten, dass einerseits altersbedingte Verluste hochaltrige Menschen, deren Familien sowie die Gesellschaft vor Herausforderungen stellen, und andererseits verbleibende Potenziale im Alter zugunsten anderer eingesetzt werden können und sollen. Die Ansichten der Stakeholder unterschieden sich jedoch nach dem Ausmaß ihres beruflichen Kontakts zu (sehr) alten Menschen. Es wurden Wege vorgeschlagen, über die die beobachteten Stereotype und Determinanten von EGW miteinander verbunden sein können (z. B. Internalisierung von Stereotypen).

**Diskussion:**

Die Studie verdeutlicht die Relevanz externaler Standards für individuelle LQ und betont die Notwendigkeit einer normativen Perspektive in der Diskussion um LQ und deren Förderung.

## Background

As “one of the most salient social categories” [51, p. 477], age activates a variety of societally shared beliefs about older people, their attributes, behavior, competencies and desires, also referred to as *old age stereotypes*. By exaggerating the extent to which older people resemble each other, stereotypes both homogenize them and separate them from other social groups [4]. It has been shown that individuals in all age groups hold both positive and negative stereotypes of older people [20]. Positive stereotypes depict them, for example, as warm-hearted, loyal, or reliable [3]; however, negative stereotypes have been repeatedly found to predominate [2, 3, 27, 44, 51]. Common negative stereotypes about older people include their suffering from poor health and loneliness, physical and cognitive incompetency, unproductivity and unattractiveness [2, 3, 9, 44].

Previous research reported adverse effects of age stereotypes on developmental outcomes including physical and cognitive performance [24], objective as well as subjective health [26, 52], and mortality [25]. Old age stereotypes also influence behavior, expectations, and judgements of people towards older individuals [2, 3, 27]. Due to ageist attitudes, “older people may be judged as inferior to middle-aged adults in terms of power and social status, wealth, respect, and influence” (age-based social status, [47, p. 650]). Perceptions of a typical older person being disengaged from “positions associated with prestige and respect” [38, p. 531] have led to an image of older people having no active or necessary role in society [15], even posing an economic burden on society [29]. While this deficit-oriented perspective has been challenged by a number of positive aging models that emphasize the potentials of older people, such as *successful* [39], *active* [17], or *productive* aging [6], critics argue that these concepts include all the more prescriptive stereotypes. By posing demands on older people to contribute, they put those with lacking options or motives to do so at risk of negative judgement (e.g., [46]).

In line with the distinction between inner valuation and external worth (utility) of an individual’s life as two kinds of life results [48], we expect external evaluations to constitute a crucial part of older individuals’ quality of life [31, 48, 50]. Building the framework of our paper, the challenges and potentials (CHAPO) model of quality of life of the very old [50] considers societal standards a resource for or threat to individual quality of life (QoL). Correspondingly, one’s perceived recognition and position within society is regarded as an individual life result [50]. Specifically, it targets very old individuals’ perceptions of being needed by society vs. being treated as a burden to society. Feeling needed has been shown to be a crucial part of purpose in life [8] as well as positive life orientation [45], both being linked to mortality. Moreover, literature on perceived burdensomeness reports that it is related to suicidal ideation severity as well as suicide attempt history [7].

In today’s western societies, with their strong orientation towards youth and performance [2], people in very old age, as distinguished from the so-called *young old* [32], are expected to be particularly confronted with lacking societal acceptance [11]; however, there are no representative analyses of perceived societal appreciation in very old age, and individual factors contributing to (not) feeling societally appreciated in very old age remain unclear. Against the background described above, the stereotypes about very old people prevailing in their environment are a promising contextual information for a more thorough understanding of perceived societal appreciation in very old age and its predictors. Therefore, the aim of the current paper is threefold:

First, the present article analyzes the subjectively perceived societal appreciation (PSA) of people in very old age (VOA) in the German federal state of North Rhine-Westphalia, as well as individual factors predicting PSA. Due to the prescriptive stereotypes promoted by current concepts of positive aging and the stigma attached to older people’s alleged inability to contribute to the economic power of the country [47], we expect individuals to feel more appreciated if they lead an *active or engaged life* (e.g., volunteering). In addition to aspects of their current lifestyle, we are interested in whether *past achievements* (child education; social status as a result of education and professional biography) are similarly significant to PSA in very old age. Lower socioeconomic status has been found to be associated with experiences of age discrimination [14, 37]. Moreover, we expect *health-related indicators* (e.g., number of chronic diseases, cognitive status, care dependency) to be crucial to PSA as they co-determine options of engagement and are known to carry a discernible stigma [41]. Functional limitations have also been found to be related to experiences of age discrimination [14]. In light of the double standard of aging (e.g., [5]) and the fact that women have been shown to be more likely to attribute experiences of discrimination to their age than men [14], we consider *gender* as another possible predictor of PSA. Finally, we consider* age*, which should not only intuitively be an important predictor but has been shown to be the primary driver of perceived age discrimination [13].

Second, we describe stereotypes about people in VOA held by stakeholders from key societal areas in North Rhine-Westphalia. Due to their position, they contribute to the normative age climate, and very old people’s opportunities within their environment.

Third, the present paper discusses the results of both studies and their possible connections. To our knowledge, this is the first study to use information on the societal values towards very old people and very old people’s perceived appreciation by society to complement each other as an example of the relation between external standards and individual life results, according to the CHAPO model.

## Material and methods

This mixed methods study used data from the survey “Quality of Life and Well-Being of the Very Old in North Rhine-Westphalia (NRW80+)”. Following a parallel design [21], quantitative and qualitative data were first analyzed independently. Subsequently, results were juxtaposed for comparison and for an extended picture of the potential connections between the societal appreciation perceived by people in VOA, and societal stereotypes about people in VOA.

### Quantitative study

Quantitative analyses used representative data of people aged 80 years and older residing in Germany’s most populated federal state of North Rhine-Westphalia (*n* = 1863). The sample covers persons living in both private and institutional settings. To allow for valid cross-group comparisons, men and those belonging to older age groups (85–89 years, 90+ years) have been oversampled. A total of 176 interviews were conducted with proxy informants when target persons were unable to participate themselves due to severe health issues. For detailed information on the study design and sample, see [16].

#### Independent variables.

The *number of treated health conditions *was measured applying an extended version of the Self-administered Comorbidity Questionnaire [40], which presented participants with a list of 19 medical conditions and asked them whether they were currently being treated for them. *Cognitive status* was measured applying the DemTect [19] in target persons, and the Global Deterioration Scale [36] in proxy interviews. Based on respondents’ last occupation, *social status* was coded according to Ganzeboom [12] with values ranging from 16 (e.g., cleaning staff) to 90 (e.g., judges). Information on further independent variables can be found in the electronic supplement (appendix A).

#### Dependent variables.

Two questions referring to discrete dimensions of PSA were used: “Do you feel needed by society?” and “Do you feel treated as a burden to society, e.g., due to physical impairments?” Response options were given as a scale from 1 = strongly disagree to 4 = strongly agree. Respondents were instructed to answer these questions based on their perception of the general societal behavior towards very old people instead of that of their closer social environment.

#### Statistical analyses.

First, we calculated descriptive statistics of all variables. χ^2^-tests (for categorical variables) and Kruskal-Wallis tests (for continuous variables) were used to examine differences in PSA by independent variables. Linear regression models were conducted to predict the two dimensions of PSA by independent variables. All analyses were performed with weighted data by use of IBM SPSS software version 27 (IBM Corp., Armonk, NY, USA).

#### Multiple imputation.

Full information on missing values is given in the electronic supplement (appendix B). As the share of missing values in cognition was 16.8%, the multiple imputation function of SPSS 27 was used to generate 5 imputed datasets in a linear procedure using all the abovedescribed variables.

### Qualitative study

Data on stereotypes about people in VOA were collected in semi-structured interviews. Stakeholders (*n* = 22) holding executive positions in key societal areas were interviewed. To capture the perspectives of an extensive range of societal stakeholders, representatives of different areas were sampled. They worked in the fields of:

- politics (*n* = 5): national *(pol-nat)* and federal state level *(pol-fed)*
- mobility management (*n* = 1)* (mobil)*
- media (*n* = 2): public *(med-pub)* and private transmitters *(med-priv)*
- senior citizen interest groups (*n* = 2) *(intgr)*
- senior citizen community services (*n* = 4): community foundations *(serv-comm)* and umbrella organizations *(serv-umb)*
- healthcare provision (*n* = 4): service providers *(hcprov-serv)* and instructors *(hcprov-instr)*
- healthcare regulation (*n* = 4): statutory *(hcreg-stat)* and private health insurances *(hcreg-priv)*, medical review board of the statutory health insurance funds *(hcreg-medrev)*, Medical Association *(hcreg-ma)*

Interviews lasted between 60 and 115 min. They were guided by two sets of questions referring to interviewees’ contact with people in VOA in their everyday work as well as their perceptions of people in VOA (see appendix C in the electronic supplement).^1^ All interviews were recorded and fully transcribed (except for one due to the interviewee’s consent to written documentation of the interview only).

The coding procedure followed methods of qualitative content analysis [22]. Inductive coding was carried out by four investigators independently supported by the MAXQDA Analytics Pro 2018 software (VERBI, Berlin, Germany). Codes were revisited and revised within the team of investigators applying criteria of appropriateness and clear distinction of codes. In this way, a consensual category system was developed. Based on this system, each interview was first analyzed individually. This was followed by a generalizing analysis [23] to look beyond the single cases and develop more general conclusions: Similarities and disparities between interviews were identified to gain an impression of the positions that seem typical for some or all interviewees. These general trends were interpreted with reference to the respective single cases.

## Results

### Quantitative analysis

Appendix D in the electronic supplement shows descriptive sample characteristics and bivariate analyses. Of the total sample 59.9% felt rather not or not at all *needed by society* and 13.8% felt rather or strongly *treated as a burden to society*. These feelings differed significantly by nearly all independent variables.

Table 1 shows results of linear regressions. They confirm that a stronger feeling of being needed by society was predicted by younger age and better cognitive health but even more so by a higher frequency of given instrumental and emotional support, club or organization membership, and volunteering. Surprisingly, given financial support and family caregiving had negative effects on the feeling of being needed by society. The feeling of being treated as a burden to society was primarily predicted by health-related variables: individuals with more treated health conditions, in full in-patient care, and dependent on care felt more treated like a burden. Club or organization membership and given instrumental support had a favorable impact, while a higher frequency of given emotional support was related to a higher feeling of being treated as a burden.

**Table 1:** Linear regression models predicting dimensions of perceived societal appreciation (*n* = 1863)

	Feeling needed by society	Feeling treated as a burden to society
	® (SE)^a^	® (SE)^a^
Constant	3.0 (0.55)***	1.89 (0.46)***
Age	−0.06 (0.01)*	−0.04 (0.01)
Gender	0.04 (0.05)	−0.02 (0.04)
Number of treated health conditions	−0.04 (0.20)	0.22 (0.16)***
Cognition	−0.09 (0.04)***	0.05 (0.04)
Care dependency	−0.05 (0.07)	0.09 (0.05)**
Full in-patient care	−0.04 (0.09)	0.12 (0.07)***
Social status	−0.02 (0.00)	0.00 (0.00)
Parenthood	0.03 (0.07)	−0.01 (0.06)
Club/organization membership	0.13 (0.06)***	−0.08 (0.05)**
Volunteering	0.13 (0.08)***	−0.03 (0.06)
Further education	0.02 (0.05)	0.00 (0.07)
Family caregiving	−0.05 (0.09)*	0.01 (0.08)
Financial support	−0.06 (0.05)**	0.00 (0.04)
Emotional support	0.09 (0.02)***	0.07 (0.02)**
Instrumental support	0.18 (0.02)***	−0.07 (0.02)*
*R*_*adj*_^*2b*^	0.192***	0.115***

*® (SE)* standard error, *R*_*adj*_^2^ adjusted R-squared

Weighted data. Results obtained from imputed dataset. Information on differing results retrieved from the original data is provided in the electronic supplement (app. E). *p ≤ 0.05 **p ≤ 0.01 ***p ≤ 0.001

^a^Coefficients and standard errors calculated as mean values from all imputed data sets as SPSS does not support calculation of values for the combined dataset

^b^Calculated as the mean value of *R*_*adj*_^2^ of all imputed data sets

### Qualitative analysis

Based on the categories derived from the qualitative interviews, four overarching themes of stereotypes about people in VOA emerged. Within them we found similar, but also differing perceptions. Differences occurred mainly between two groups of stakeholders, with one group comprising those whose profession primarily included direct contact with (very) old people (G1: senior citizen community services, health care providers, senior citizen interest groups) and a second group consisting of those whose work did not (G2: politics, mobility management, media, healthcare regulation).

#### Losses

A wealth of perceptions of very old people expressed by interviewees revolve around losses in different areas of life.

##### Health and independence.

All stakeholders refer to people in VOA as a fragile group characterized by multimorbidity, cognitive decline, and care dependency:* “generally, they all have some kind of impairments” (hcprov-serv). *These are expected to determine very old people’s lives: After an incisive event leading to care dependency, *“nothing is like it was before” (pol-fed)*; illness, pain, or dementia *“shapes everyday life. It shapes the whole day” (hcprov-serv)*.

##### Social contacts.

As many very old people had to witness the death of peers, *“they lost so many people on the way” (intgr)*, they are seen as restricted in their social life and at risk of isolation.

##### Societal recognition.

Stakeholders regard people in VOA to be little-noticed or even ignored by society: *“We don’t know about [them] because we look away” (serv-comm), “they don’t have a voice” (mobil)*.

##### Financial security.

Stakeholders mention the threat of old-age poverty, which they consider particularly high for very old women due to their limited opportunities to receive an extensive education and to pursue a paid profession in the past.

Due to losses in health or functionality, interviewees from G2 think of very old people as imposing a burden on their families in the sense of healthcare costs, informal care, or the difficult decision regarding potential institutionalization. On a societal level, they see challenges arising due to very old people’s lack of cognitive flexibility to adapt to changing societal perspectives. Furthermore, they express worries about the national economy: *“Economically, it’s of course highly problematic, because we have too few young ones who can pay for the very old one [s’ healthcare costs]” (pol-nat)*. They also draw on economic reasons when speaking about the lack of very old people’s societal inclusion: A stakeholder from mobility management refers to them as a group of customers that is *“unattractive” *to design specific services for as the very old will not use such services *“sustainably”.* Stakeholders from media argue that showing older people or addressing age-relevant themes on television is something* “you can’t allow yourself” (med-pub), *especially as private transmitters, as younger audiences are the *“currency” (med-priv) *in the advertising industry.

Interviewees from G1, in contrast, are aware that loss-related associations fail to represent the overall very old population: *“One cannot say that someone, just because they are 80, suffers from dementia” (hcprov-serv)*. Yet, they perceive very old people as threatened by age-discrimination due to such misconceptions. They deplore very old people being *“pushed aside” (intgr)* by arbitrary age limits, for instance, in (voluntary) work or empirical research. Moreover, they protest that care-dependent persons are too often *“shunted”* into a nursing home and then *“left alone*” *(serv-comm)* by their families.

#### Experiential richness

All stakeholders are convinced that people in VOA possess a great wealth of experiences. Besides huge general, historical, and professional knowledge, very old people are perceived to have developed specific skills and strategies by surviving times of war, escape, and reconstruction. They are characterized by stamina as well as abilities to manage with little and adapt to losses. There is agreement that drawing on such resources, people in VOA take on responsibilities within society and provide valuable support to younger generations. Nevertheless, stakeholders from G1 view very old people’s resources as relevant primarily for themselves and their personal development: *“They have come so far (...), they have everything within themselves”* (*serv-comm)*. They explicitly point to resources also in those with health restrictions: Even persons with dementia are able to experience individual achievements and *“true inner happiness” (serv-comm), *and because of their specific view of life, they *“show what’s the here and now” (serv-comm)*. Stakeholders from G2, in contrast, more often speak of *“a very big wealth of experiences that can be passed on” (mobil), *and link remaining resources to staying engaged.

#### Otherness

Another broad conception of people in VOA is that they in some sense differ from the rest of society. Stakeholders from G1 attribute this to changing perspectives of life: in old age, *“life (...) in its entirety may be construed differently”* (*hcprov-serv*). This includes a reinvention of one’s life and a stronger concentration on oneself as a result of biographical disruptions, such as the death of a spouse. From the outside, they find this age-related change in perspectives is often misconceived as social withdrawal. Stakeholders from politics and media, in contrast, rather refer to very old people’s past socialization, which leads them to believe that people in VOA hold values, attitudes, and an image of society (e.g., the role of women) that are not consistent with modern perceptions. For example, some stakeholders of G2 think of people in VOA as *“completely refusing (...) to even try” (med-priv) *using modern technologies. In contrast, stakeholders from G1, but also one stakeholder from healthcare regulation, stress that by not using digital technologies, people in VOA preserve qualities such as patience that younger generations are losing. They see very old people in a dilemma between societal demands to use modern technologies in order *“not to be left behind”* (*intgr*) and having no intrinsic motivation to do so.

#### Heterogeneity

Although people in VOA are associated with a number of specific characteristics as presented in the previous sections, stakeholders from G1, and one stakeholder from politics describe them as a very heterogeneous population. This is attributed, on the one hand, to markedly different experiences and life chances between cohorts (e.g., those born before, during, or after World War II). On the other hand, interindividual differences do not disappear *“just because it was one’s birthday” (hcprov-serv)*:* “A person does not turn into another one just because of old age” (pol-fed)*. Basically, people in VOA are viewed as equally heterogeneous as younger groups.

## Discussion

In our quantitative study, we found that PSA was primarily predicted by health-related variables along with aspects of an engaged lifestyle. Other aspects of the person and their biography were not shown to be relevant in the multivariate analysis, indicating that older people’s perceived external appraisal depends on their current condition and performance, irrespective of their previous path of life. Gender not showing a significant effect on PSA could be explained by the fact that the model controls for level of engagement, as traditional role expectations demand more caring behavior from women than from men. This possible interaction effect was not investigated in our model and could be a matter for future research on societal appreciation in very old age; however, not all productive activities had positive effects. Financial and emotional support as well as family caregiving each had a negative effect on one of the PSA dimensions. This might be explained by emotional support being perceived as less *measurable* or *profitable* than instrumental, and financial support as well as caregiving being in many cases unavoidable or concealed acts within the closest family. The adverse impact of caregiving is in line with abundant evidence of caregiver burden and various negative consequences of family caregiving [1].

The qualitative analysis showed that societal stakeholders shared similar overarching perceptions of people in VOA, but differed in more detailed perceptions, and in how they contextualized and interpreted certain perceptions. In general, stakeholders working in direct interaction with (very) old people drew a more differentiated picture of VOA-related losses (e.g., adverting to positive implications of losses), and expressed a more positive view on very old people. They showed higher awareness of the impropriety and danger of negative stereotypes about people in VOA, and repeatedly pointed to shortcomings in societal treatment of very old people. In contrast, stakeholders whose work did not primarily include direct contact with (very) old people tended to adopt a societal perspective. They primarily saw disadvantages in very old people’s *otherness*, addressed challenges and burdens arising with population aging, and tended to expect that very old people’s remaining resources are, and should be, used for the benefit of others. The observed differences between these groups of stakeholders are consistent with previous research reporting that closer contact to older people leads to more differentiated, and positive beliefs of them [3].^2^ Moreover, it is striking that since the official identification of younger audiences as the advertising-relevant target group by a German private transmitter in the 1990s [30], little has changed about the industry’s perceptions of older people despite an aging society. This result is in line with various content analyses reporting underrepresentation of older people in the media (e.g., [28]).

The views of the latter group of stakeholders mirror the relevance of physical and functional health and an engaged lifestyle to PSA found in our quantitative study. There are several pathways through which this correspondence might come about.

First, individuals are expected to be aware of the stereotypes about them held by others, and to rely on these when predicting the opinion of others about them [10, 34, 49]. Hence, our results about determinants of PSA imply that very old people perceive less differentiated and more negative stereotypes as more representative of societal notions. This is consistent with numerous findings that negative stereotypes prevail in western societies [2, 3, 27, 51].

Second, stereotypes lead to certain behavior culminating in stigmatization and discrimination [2, 27]. Observing verbal and nonverbal behavior of others helps to determine how one is viewed by them [10]. Against this background, our results indicate that those with more health-related losses and those unable or unwilling to engage in certain activities experience worse societal treatment. This highlights that age-related stereotypes and their manifestations are most often not only ageist, but also ableist. They consider older people to be of less value because they are sick, dependent, and therefore unproductive [18]. Norms of achievement and social utility, which are typical for midlife, are projected on the last phase of life, with discriminatory effects on those who do not or cannot fulfil them [11, 18, 46].

Finally, there is overwhelming evidence that through processes of internalization, individuals integrate stereotypes held about them into their self-image [3, 51, 53]. Being another source for estimating how one is seen by others, an adverse self-image due to exposure to negative stereotypes will reinforce the expectation to be judged negatively [10]. Moreover, internalized beliefs influence older people’s behavior and, in turn, reinforce others’ perceptions of them. This also points to the concept of “doing age”, which describes age as a social construction resulting from a process of social ascriptions and interactive demonstrations, which influences the image of older people held by others, but also held by older people themselves [42].

Summing up, societal attitudes towards the very old population and perceived societal appreciation in VOA might relate to each other in different ways that are not mutually exclusive, but likely interdependent. This illustrates the relevance of external standards and normative stipulations for individual QoL as presumed by the CHAPO model of QoL of the very old [50], and highlights the need for a normative perspective in the discussion about QoL and possible measures for its enhancement.

### Limitations and future research

In our qualitative study, we sampled representatives of markedly different areas. Although this might seem problematic for the drawing of overarching conclusions, a heterogeneous sample captures a wide range of perspectives on the topic of interest and is therefore an important strategy to approach conceptual (not statistical) representativity (maximum variation sampling, [43]). We believe that the heterogeneity of our sample was necessary to capture the perspectives of an extensive range of societal stakeholders in order to identify common themes but also uniqueness that differentiate them from each other [43].

Moreover, we did not calculate intercoder reliability (ICR) in our qualitative study, although it is frequently recommended and can enhance rigor and transparency of the coding frame; however, its use is not uncontroversial among qualitative researchers nor appropriate for every qualitative study (for a synopsis of arguments in favor of and against ICR, see [33]). In our case, the calculation of ICR did not seem reasonable as intersubjective congruence in the explorative, consensual construction of categories cannot be postulated [35, p. 102–103]. Rather, the process gains from discussion between coders, which ensures the tapping of the full creative potential of the team [33, 35].

Note that our study design naturally did not allow for causal conclusions. Nonetheless, we aimed to give some thoughts about the possible connection of the observed stereotypical beliefs and very old people‘s perceived societal appraisal. Following this initial approach to merging societal and individual perspectives on very old people and their value in society, future research could further entangle both levels, e.g. by considering individuals’ different degrees of exposure to relevant societal forces.

## Practical conclusion

- Stereotypes about very old people are relevant to individual quality of life in very old age.
- Stereotypes need to be reduced, e.g., through educational initiatives at schools, intergenerational or community-based mentorship programs, realistic portrayals of older people in the media, or by installing an official political position counteracting them.
- The public should be sensitized to the manifestations and consequences of stereotypes and encouraged to interfere when witnessing them.
- Older individuals should be educated about mechanisms of stereotype internalization, empowered to protect their self-image, and encouraged to stand out against mistreatment.

## Supplementary Information

Further independent variables, information on missing values, guiding questions for qualitative interviews, descriptive characteristics and bivariate analyses, addition to table 1.

## Footnotes

1 To avoid confoundation with interviewees’ solely private experiences with older people, the guiding questions repeatedly addressed them as representatives of their professional position, and referred to their experiences with (very) old people within their professional environment.

2 As described in the methods section, the duration of interviews varied between 60 and 115 min. There was, however, no systematic difference in duration of interviews between G1 and G2 (95%-CI = −8.98–27.81, t(20) = 1.07, *p* = 0.298). More differentiated or in-depth answers were thus not a result of longer interviews.

## Declarations

All procedures performed in studies involving human participants or on human tissue were in accordance with the ethical standards of the institutional and/or national research committee and with the 1975 Helsinki declaration and its later amendments or comparable ethical standards. Informed consent was obtained from all individual participants included in the study.
