# Supplementary material for: Stereotypes about very old people and perceived societal appreciation in very old age
Source: Z Gerontol Geriatr. 2021 Oct 1;54(Suppl 2):93–100. doi: 10.1007/s00391-021-01971-y (PMC8551093; doi:10.1007/s00391-021-01971-y)
Supplement: Supplementary file 1 — Further independent variables, information on missing values, guiding questions for qualitative interviews, descriptive characteristics and bivariate analyses, addition to table 1. [file 391_2021_1971_MOESM1_ESM.docx]

***Appendix A.* Further independent variables.**

We considered *age* and *gender* (male/female)*. Care dependency* was derived from respondents’ information about their care level. Additionally, respondents were asked whether they used *full in-patient care*. *Parenthood* was measured by asking study participants if they have or had any biological or foster children. Finally, respondents reported whether they were an *active* *member of any club or organization*, engaged in any *volunteer work*, *further education,* or *family caregiving*, if they had given any *financial support* to somebody during the previous 12 months, and how frequently they had given *emotional* or *instrumental support* (1=never, 5=always).

***Appendix B.* Information on missing values.**

Nearly all variables had a share of missing values ranging between 0.0% and 4.1% (med=0.2%). Informal care had 13% missing values, most of which were intentional as respondents in full-inpatient care were not asked whether they provided any informal care. Assuming that individuals in full in-patient care were not providing informal care, we coded them as “no,” which reduced the amount of missing values in this variable to 0.3%. However, the share of missing values in cognition was 16.8., which made it necessary to impute missing data as described in the paper.

***Appendix C.* Guiding questions for qualitative interviews.**

| **Block 1: Questions about stakeholder functions** | |
| --- | --- |
| **To what extent do you come into contact with questions of demographic change and the living conditions of older and very old people in your current functions?** | |
| **Content aspects** | **In-depth questions** (optional) |
| \| Interfaces with old people \| \| --- \| | \| - What specific points of contact do you have in your work with older people? - What role do older people play in your work compared to younger generations (adolescents; young adults)? \| \| --- \| |
| Group of the elderly | \| - Are there certain groups of older people who are particularly relevant to your work? - How do you characterize this group of older people? \| \| --- \| |
| Old age | - Which of your work or activities are explicitly aimed at very old people (over 80 years of age)? - Who predominantly represents the interests of the very old towards you? |
| \| Structure / institution / facility \| \| --- \| | \| - What options do you have in shaping and making decisions based on your position in this institution / facility? \| \| --- \| |

| **Block 2: Questions about (very) old age** | |
| --- | --- |
| **When you hear the term “very old”, what do you think of spontaneously? What do you associate with this term?** | |
| **Content aspects** | **In-depth questions** (optional) |
| THE very old person or THE old person | - How would you describe the very old / old person prototypically / stereotypically? - Where do you encounter stereotypes about age / very old age in everyday work / in your function? - To what extent does your view of the elderly coincide with clichéd ideas about old age? |
| Social position of the very old / old | - Based on your function, how would you describe the positions of older and especially very old people in our society (e.g. cost or economic factor, protected object)? - To what extent do you see the conditions in our society in order to appreciate older people with their life achievements and their way of being? |
| Needs of the elderly | - What special needs do you see of older and very old people? - To what extent can you address this through your work? - What opportunities do you see for improvement in order to respond more specifically to the needs of older people in our society? - To what extent do you see a need to implement certain protective regulations (e.g. "rights of the elderly", separate penalties) for older people? - How do you see the relationship between the needs of older generations and younger generations? |
| Quality of life in old age | - What role does the topic of "quality of life" play in your day-to-day work / in your role? - What ideas about the good life in old age determine how you deal with the quality of life in everyday working life? |
| Skills of the Elderly | - What special potential for society do you see in the skills and experience of older people? - To what extent can you explicitly address these skills through your work? - What differences do you see between older and younger generations in terms of socially relevant skills? |
| Future goals | - From your perspective, what specific goals should be pursued in the future with a view to the very old population? - In which social fields and areas do you see a particular need for action in the future in order to promote the quality of life of the very old? |

*Note*. The questions had guiding character for the interview. In-depth questions served as optional/exemplary questions to be asked in the narrative interview.

***Appendix D.* Descriptive characteristics and bivariate analyses by dimensions of perceived societal appreciation (PSA) (n=1863).**

|  | | **Feeling needed by society**  mean (SD) / percent | | | | |  | **Feeling treated as a burden to society**  mean (SD) / percent | | | | |
| --- | --- | --- | --- | --- | --- | --- | --- | --- | --- | --- | --- | --- |
|  | | *Strongly disagreed* | *Rather disagreed* | | *Rather agreed* | *Strongly agreed* | *p value^a^* | *Strongly disagreed* | *Rather disagreed* | *Rather agreed* | *Strongly agreed* | *p value^a^* |
|  | | 34.5% | 25.4% | 25.5% | | 14.6% |  | 65.0% | 21.2% | 10.0% | 3.8% |  |
| Age | | 85.8 (4.2) | 84.7 (4.0) | 84.2 (3.9) | | 83.6 (3.3) | <.001 | 84.5 (3.9) | 85.7 (4.2) | 85.1 (4.0) | 84.7 (3.7) | <.001 |
| Gender | female | 69.4% | 63.9% | 64.2% | | 69.0% | .152 | 65.6% | 67.6% | 69.9% | 70.2% | .573 |
|  | male | 30.6% | 36.1% | 35.8% | | 31.0% |  | 34.4% | 32.4% | 30.1% | 29.8% |  |
| Number of treated health conditions | | 1.9 (1.3) | 1.8 (1.1) | 1.9 (1.2) | | 1.6 (1.2) | .008 | 1.7 (1.2) | 1.9 (1.3) | 2.4 (1.3) | 2.8 (1.5) | <.001 |
| Cognition | healthy | 55.5% | 65.7% | 78.2% | | 80.5% | <.001 | 71.9% | 59.7% | 59.4% | 57.8% | <.001 |
|  | MCI | 15.6% | 18.3% | 11.9% | | 16.0% |  | 15.6% | 15.7% | 13.8% | 13.8% |  |
|  | dementia | 28.9% | 16.0% | 9.8% | | 3.5% |  | 12.5% | 24.6% | 26.70% | 28.4% |  |
| Care | yes | 50.5% | 36.1% | 28.1% | | 17.6% | <.001 | 28.2% | 47.9% | 55.4% | 59.8% | <.001 |
| dependency | no | 49.5% | 63.9% | 71.9% | | 82.4% |  | 71.8% | 52.1% | 44.6% | 40.2% |  |
| Full in-patient | yes | 20.6% | 14.6% | 7.1% | | 2.5% | <.001 | 7.7% | 21.8% | 27.1% | 16.6% | <.001 |
| care | no | 79.4% | 85.4% | 92.9% | | 97.5% |  | 92.3% | 78.2% | 72.7% | 83.4% |  |
| Social status | | 38.7 (20.7) | 40.2 (19.9) | 42.2 (20.1) | | 41.7 (20.8) | .015 | 41.2 (20.5) | 39.4 (20.5) | 37.4 (19.4) | 40.1 (20.7) | .101 |
| Parenthood | yes | 86.0% | 85.2% | 89.3% | | 92.3% | .018 | 88.9% | 84.5% | 82.3% | 95.5% | .004 |
|  | no | 14.0% | 14.8% | 10.7% | | 7.7% |  | 11.1% | 15.5% | 17.7% | 4.5% |  |
| Club/organization | yes | 14.0% | 21.8% | 31.6% | | 45.1% | <.001 | 29.8% | 17.3% | 13.4% | 15.1% | <.001 |
| membership | no | 86.0% | 78.2% | 68.4% | | 54.9% |  | 70.2% | 82.6% | 86.6% | 84.7% |  |
| Volunteering | yes | 4.7% | 9.6% | 18.9% | | 30.5% | <.001 | 15.9% | 10.7% | 3.3% | 10.3% | <.001 |
|  | no | 95.3% | 90.4% | 81.1% | | 69.5% |  | 84.1% | 89.3% | 96.7% | 89.7% |  |
| Further | yes | 5.2% | 8.1% | 12.4% | | 15.4% | <.001 | 10.7% | 6.0% | 8.0% | 6.0% | .030 |
| education | no | 94.8% | 91.9% | 87.6% | | 84.6% |  | 89.3% | 94.0% | 92.0% | 94.0% |  |
| Family caregiving | yes | 5.8% | 4.9% | 5.7% | | 7.8% | .158 | 6.4% | 5.3% | 5.4% | 4.5% | .758 |
|  | no | 94.2% | 95.1% | 94.3% | | 92.2% |  | 93.6% | 94.7% | 94.6% | 95.5% |  |
| Financial | yes | 30.2% | 34.3% | 34.0% | | 37.4% | <.001 | 35.1% | 30.5% | 28.5% | 30.2% | <.001 |
| support | no | 69.8% | 65.7% | 66.0% | | 62.6% |  | 64.9% | 69.5% | 71.5% | 69.8% |  |
| Freq. of emotional support | | 2.3 (1.2) | 2.7 (1.1) | 2.8 (1.1) | | 3.0 (1.2) | <.001 | 2.6 (1.2) | 2.6 (1.1) | 2.6 (1.2) | 2.6 (1.4) | .482 |
| Freq. of instrumental support | | 1.7 (1.1) | 2.0 (1.2) | 2.3 (1.3) | | 2.9 (1.3) | <.001 | 2.2 (1.3) | 1.8 (1.1) | 1.7 (1.1) | 2.1 (1.3) | <.001 |

*Note*. Weighted data. Results obtained from imputed dataset. Value ranges: for social status: 16 (e.g. cleaning staff) to 90 (e.g. judges); for freq. of emotional/instrumental support: 1 (never) to 5 (always); for feeling needed by society/feeling treated as a burden: 1 (strongly disagreed) to 4 (strongly agreed).

^a^ Chi square tests were used for categorical variables, Kruskal-Wallis tests for continuous variables

***Appendix E.* Addition to table 1: Differing results of linear regression models predicting feeling needed by society and feeling treated as a burden to society based on the original dataset (n=1863).**

In deviation from the results retrieved from the imputed dataset, feeling needed by society was significantly predicted by the number of treated health conditions (b=-0.05, p<0.05). Given instrumental support was not a significant predictor of feeling treated as a burden to society in the original dataset.
